# New Caledonian Crows Learn the Functional Properties of Novel Tool Types

**DOI:** 10.1371/journal.pone.0026887

**Published:** 2011-12-14

**Authors:** Alex H. Taylor, Douglas M. Elliffe, Gavin R. Hunt, Nathan J. Emery, Nicola S. Clayton, Russell D. Gray

**Affiliations:** 1 Department of Psychology, University of Auckland, Auckland, New Zealand; 2 School of Biological and Chemical Sciences, Queen Mary University, London, United Kingdom; 3 Department of Experimental Psychology, University of Cambridge, Cambridge, United Kingdom; Yale University, United States of America

## Abstract

New Caledonian crows were presented with Bird and Emery's (2009a) Aesop's fable paradigm, which requires stones to be dropped into a water-filled tube to bring floating food within reach. The crows did not spontaneously use stones as tools, but quickly learned to do so, and to choose objects and materials with functional properties. Some crows discarded both inefficient and non-functional objects before observing their effects on the water level. Interestingly, the crows did not learn to discriminate between functional and non-functional objects and materials when there was an arbitrary, rather than causal, link between object and reward. This finding suggests that the crows' performances were not based on associative learning alone. That is, learning was not guided solely by the covariation rate between stimuli and outcomes or the conditioned reinforcement properties acquired by functional objects. Our results, therefore, show that New Caledonian crows can process causal information not only when it is linked to sticks and stick-like tools but also when it concerns the functional properties of novel types of tool.

## Introduction

Chimpanzees use tools in the wild flexibly (for review see [1]). That is, tools are used in different contexts, such as foraging, grooming, and social interactions, and many different types of natural material, including stone, are involved in tool use and manufacture. In contrast, although New Caledonian crows (*Corvus moneduloides*) show sophisticated tool behaviours both in the wild [2]–[5] and in captivity [6]–[10], their natural tools are manufactured from plant material and are only used during foraging. Their tool behaviour is, therefore, much more context-specific than that of chimpanzees.

The absence of stone tool use is particularly interesting because these crows drop nuts onto hard surfaces [11], unlike apes and monkeys who do use stone tools to crack nuts. Thus there is a clear and valid ecological context for the development of stone nut-cracking tools in New Caledonian crows. Furthermore, other bird species have developed stone tool use. In the wild, Egyptian vultures throw stones onto ostrich eggs [12]. In captivity, two otherwise non-tool using species, rooks and Eurasian jays, use stones to obtain out-of-reach food and can learn to discriminate between functional and non-functional objects and substrates [13]–[15].

The lack of stone tool use by free-living New Caledonian crows could be because the cognition underlying their tool use is highly domain-specific. That is, while these crows are able to grasp the functional properties of sticks and stick-like objects, they cannot understand the properties of novel objects that have no connection to their established repertoire of wild behaviours. Recent evidence supports this hypothesis – when presented with string, New Caledonian crows struggled to solve string pulling tasks when visual feedback was restricted and failed the crossed string connectivity task [16]. Similarly, while these crows appeared to be sensitive to the interaction between meat, stick tool and hole when solving the trap-tube problem, they were insensitive to the interaction between meat and the trap-base [17], [18]. That is, they learnt to avoid pushing meat into holes with their tools but did not realise that food would pass through a hole without a base. Perhaps, then, this species has what Sterelny refers to as a ‘narrow-banded ability’ to process causal information [19]. While these crows are exposed to causal information about many agents, objects and contexts in the world, they may only process causal information that relates to stick-like objects. That is, there may be only a narrow range of object-object interactions in the world (all involving stick-like objects) that the crows can understand causally. This would still allow the crows to be creative and flexible in their tool use, while ignoring potentially distracting causal interactions that are not directly relevant to survival.

We can directly test this hypothesis by exploring New Caledonian crows' ability to use and understand the properties of stone tools. In the one study reported to date [20], New Caledonian crows were presented with a collapsible platform apparatus originally used with rooks [14]. Although the crows did not spontaneously drop stones down a tube to collapse the platform and release food, two individuals trained to nudge stones down the tube from a ledge solved the problem. Another four birds were trained to push down the platform with their bills, and two of these then spontaneously picked up and dropped stones down the tube despite having never observed this behaviour or used stones as tools. This suggests that learning how the interaction involved in the task unfolded – that the platform collapsed if contact was made with it – was sufficient to allow the crows to solve this task, despite their lack of experience using stones as tools. However, it is unclear from this experiment whether the crows understood anything about stones beyond the fact that they were objects that could be used to make contact with the platform. The crows may have used stones because they were the closest available objects, not because they understood that stones were heavy and so could collapse the platform. Consistent with this possibility, one of the two successful birds dropped a small feather into the tube between its first and second successful trials.

Here, we presented New Caledonian crows with Aesop's fable paradigm, a task which requires stones (or similar objects) to be dropped into a water-filled tube in order to raise the water level and bring floating food within reach. This allowed us to directly test whether this species can learn about the functional properties of stones and similar objects when using them as tools.

## Materials and Methods

### Ethics Statement

Our work was carried out under University of Auckland Animal Ethics Committee approval R602.

### Subjects

We carried out the experiment with five wild crows captured on the island of Maré, New Caledonia. Three of the crows (Caesar, Laura and Bess) were adults more than 2 years old and two (Mimic and Pepe) were sub-adults less than 2 years old. Based on sexual size dimorphism [21], Laura and Bess were female. The crows were housed in a five-cage outdoor aviary close to the location of capture; the cages varied in size but were all at least 8 m^2^ in area and 3 m high. Caesar, Laura and Pepe completed the entire series of experiments. Bess was replaced by Mimic after Experiment 1 because of a neophobic reaction to the experimental apparatus. After learning to stone-drop, Mimic participated in Experiments 2–6 and 9–10, but did not take part in Experiments 7 and 8. Again, this was due to a neophobic reaction. All crows were released at their site of capture after testing.

### Materials

**Figure 1:**
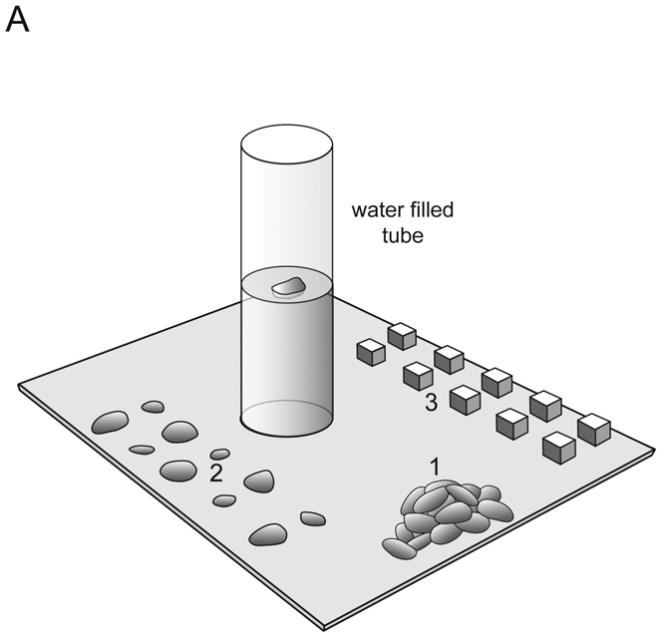
The experimental apparatus, with objects positioned next to it. (1) The presentation of stones in Experiments 1 and 2, (2) The presentation of stones in Experiment 3, and (3) the presentation of heavy and light objects in Experiment 6.

The vertical, clear glass tubes used for the stone dropping tasks were 180 mm high and 50 mm in diameter (Figure 1). Large stones weighed 14 g and small stones 2 g. Polystyrene and rubber blocks were of the same size and colour and weighed 0.25 g and 16 g, respectively. During the search experiments, the tubes where food was hidden across trials were the same size (50 mm in diameter and 70 mm long), shape and colour. For the unrelated tool discrimination tests in Experiments 9 and 10, crows were presented with a crevice made of two Perspex sides (100 mm long×70 mm high×10 mm thick) that were positioned 12 mm apart. The crows could chose between three white tools in Experiment 9 that were 80 mm long and 4 mm in diameter and made of different materials (a 6 g length of metal, a 0.4 g length of plastic and a 0.4 g length of string). In Experiment 10, the crows could choose between two white tools 80 mm long, one with a diameter of 0.4 mm weighing 0.6 g and the other with a diameter of 0.7 mm weighing 1.2 g).

### General procedure

The crows were tested in visual isolation from other crows. Trials began when a bird flew down to the table to investigate the apparatus and ended after food retrieval or 5 minutes. Crows varied in the distance that they could reach into the tube with their bills to obtain food. To ensure food was the same distance out-of-reach for each crow, we initially presented each crow with meat floating at differing heights until the ‘reachable height’ had been established. This was the lowest height at which the crow could remove the meat from the tube with its bill.

Once this height had been established, four crows (Caesar, Laura, Pepe and Bess) were first given five trials, each of 3 minutes duration, with the original Aesop's fable paradigm, to see if they would spontaneously drop stones into water (Figure 1). These four crows were then given ‘shaping’ trials to teach them to drop stones. At this point Bess had a neophobic reaction to the apparatus and was replaced with Mimic, who was also given shaping trials. Once the crows had learnt to drop stones into the tube they were given the three tasks reported in [13]: matching the number of stones to distance to water (which tested whether the crows' action was goal directed), discriminating between large and small stones (which tested whether they were sensitive to the functional properties of the objects involved) and discriminating between sand- and water-filled tubes (which tested whether they were sensitive to the functional properties of the material in the tube). As in [13], crows were given 20 trials with each of these conditions. For these experiments and Experiment 5 and 6, a stone-drop/object-drop was defined as the selection of a stone or object from the table and the dropping of the same stone or object into the tube. The crows were then given two further tests of 20 trials (as in [15]) that examined their understanding of the functional properties of the objects in the experiment. Experiment 5 examined if the crows could discriminate between water and air, and Experiment 6 examined whether the crows could discriminate between heavy and light objects of the same size and colour. Experiment 6, therefore, tested if the crows understood that objects needed to be both large and heavy in order to raise the water level substantially.

Three crows were then given two experiments where they had to search for food hidden in one of two tubes that were next to either the functional or non-functional stone (Experiment 7) or functional and non-functional tube (Experiment 8) (Mimic had a neophobic reaction to the tubes and could not be tested). The crows were given 20 trials with each experiment. These search paradigms allowed us to test whether an arbitrary link between object and outcome, rather than a causal one involving stone dropping, would lead to the same level of performance as in the object and tube choice problems (Experiments 3–6). The findings in Experiments 3–6 could be explained by associative learning if the crows were capable of associating an object with an outcome within one or several trials. That is, when a crow was successful the object involved acquired positive hedonic value, and when the crow made errors the object acquired negative hedonic value. Thus the initially neutral objects involved in the experiments would have become conditional reinforcers or punishers depending on whether the crow was successful or not. In effect, the crows would be following a simple heuristic – ‘always choose what worked before’. The search paradigms in Experiments 7 and 8 acted as an associative learning control because these experiments were essentially re-runs of Experiment 3 (large stone/small stone discrimination) and 4 (water-filled tube/sand-filled tube). The only difference in these experiments was that there was an arbitrary link between object and outcome, rather than a causal link. For example, in Experiment 3 the large stones affected the outcome by substantially raising the water level. Thus, there was a reason why the stones were positive stimuli: they efficiently raised the water level, unlike the small stones which displaced only a small amount of water. In Experiment 7, the large stone was arbitrarily linked to the outcome – there was no functional reason for it to be a positive stimulus when the crows were searching for food in tubes. Associative learning is driven by correlations between initially neutral stimuli and unconditioned stimuli or primary reinforcers (here, access to food). It should be irrelevant to a simple associative account whether these correlations are arbitrary or causal. Therefore, we made two predictions if the crows' behaviour in Experiments 3 and 4 was due to simple associative learning. First, the crows should prefer to search a tube with a large stone or water-filled tube in front of it because these objects would be conditional reinforcers due to their previous association with food in the prior experiments. Second, the crows should have a similar learning pattern – they should link the large stone and water-filled tube to success as quickly in the search paradigm as they had done in the stone-dropping paradigms.

**Figure 2:**
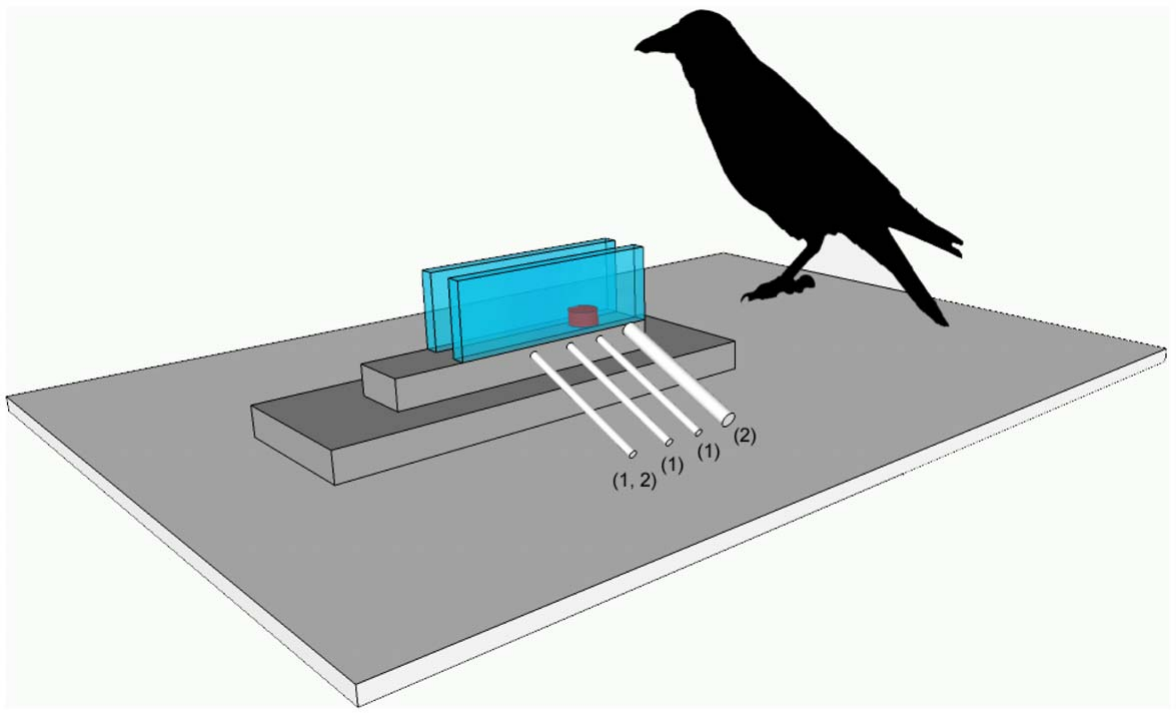
Diagram of the apparatus used in Experiments 9 and 10. (1) The tool setup in Experiment 9, and (2) the setup in Experiment 10.

In the last two experiments (Experiments 9 & 10, Figure 2), we examined if the crows' tool behaviour with sticks and stick-like objects was affected by their learning about the novel dropping tools in the previous experiments. In Experiment 9, the crows were given 20 trials where they had to choose between three tools of the same size that differed in weight and/or flexibility. In Experiment 10, they were given 20 trials to choose between two tools of the same length, one of which had a volume three times larger than the other, and a weight double that of the other. If the crows were using a heuristic, such as ‘always pay attention to the weight of objects when food is out-of-reach’, we expected them to continue to discriminate between heavy and light objects in other situations where food was out-of-reach. If the crows had an understanding of the actual mechanics of the task, we expected the transfer of knowledge of physical properties (e.g. weight) to new tasks to occur only when the mechanics of the tool use were the same. Therefore, by changing the structure of the task, but not the relative properties of the objects involved, we could examine if the crows would transfer knowledge of the functional properties of objects into situations where such knowledge was now irrelevant. That is, we could test whether knowledge about a new type of tool use involving stones and similar objects merged in a non-functional way with existing knowledge about stick tools.

### Specific procedure for each experiment

#### Experiment 1: the Aesop's fable paradigm

The water level was 12 mm beneath the reachable height and 5 stones were placed at the base of the tube. The tube was baited with a small meat block attached to a floating piece of wood. Crows were given 5 trials of three minutes duration to solve the problem. Crows that did not solve it were presented with a baited water-filled tube with a platform next to the top of the tube. Two stones were placed on this platform. When a crow attempted to reach into the tube from the platform, one or both of the stones were likely to be accidentally knocked into the tube, which made the meat attached to the wooden float move upwards and slightly closer to the crow. Testing finished once a crow had obtained the meat 10 times by dropping a stone into the water.

#### Experiment 2: matching number of stones to distance to water

Crows were presented with a baited water-filled tube with 10 stones at the base. The water level was varied in increments of 3 mm, with 7 different water levels presented three times to a crow in a pseudorandom order (no more than two trials with the same height). Each of the stones provided raised the water by 3 mm, allowing the crows to match water level to stones required.

#### Experiment 3: Object discrimination; large vs. small stones

The tube was baited and the water level was 12 mm beneath the reachable height. Crows were presented with 5 small stones and 5 large stones. These stones were arranged in a grid-like pattern with the position of small and large stones pseudorandomized across trials. Crows were given 20 trials.

#### Experiment 4: Substrate discrimination: sand vs. water

Crows were presented with two tubes placed 300 mm apart. One tube was filled with water and one with sand. Each tube contained food that was 12 mm beneath the reachable height. The position of the two tubes on the table was pseudorandomized across the 20 trials given to the crows.

#### Experiment 5: Substrate discrimination: air vs. water

This experiment was identical to Experiment 4, except that the sand-filled tube was replaced with an empty tube containing food attached by tape 12 mm beneath the reachable distance.

#### Experiment 6: Object discrimination: heavy vs. light objects

Crows were presented with a single baited tube with the water level 12 mm beneath reachable height. White rectangular blocks of rubber and polystyrene of the same size were arranged in a grid-like pattern around the base of the tube, with the position of these two object types pseudorandomized across the 20 trials given. The rubber was heavy and sinkable, but the polystyrene floated on the surface of the water.

#### Experiment 7: Searching paradigm: large stone vs. small stone

The crows had previously been given a colour learning task where they had to find food hidden in a silver tube, while avoiding searching for food in a gold tube. In this task, two tubes had been placed 300 mm apart with their open ends facing away from the crows and their closed ends facing towards the crows. The crows were allowed to fly down to the table and examine the hidden contents of one of the tubes. The meat was always hidden in the silver tube, the position of which was pseudorandomized across trials. To become proficient at this task the crows had to always search the silver tube first to get the food. In this experiment, we presented the crows with two identical grey horizontal tubes spaced 300 mm apart. As in the colour learning experiment, food was hidden in only one of these tubes in each trial, with the position of the baited tube pseudorandomized across trials. A large stone was placed in front of the baited tube and a small stone in front of the unbaited tube in each of the 20 trials given to the crows. These stones were chosen randomly from those that had been used in Experiment 3.

#### Experiment 8: Searching paradigm: sand-filled tube vs. water-filled tube

This experiment was identical to Experiment 7, except that rather than placing stones next to the search tubes, one search tube had a water-filled glass tube placed next to it and the other had a sand-filled tube placed next to it. These tubes were the same ones used in Experiment 4.

#### Experiment 9: Tool discrimination: Light vs. heavy tool

Crows had to extract out-of-reach food from a Perspex crevice. To do this they were given the choice of three white tools of the same length and diameter – a heavy and rigid metal one, a light and rigid plastic one, and a light and flexible piece of string. These tools were positioned behind the apparatus and their position was pseudorandomized across the 20 trials given.

#### Experiment 10: Tool discrimination: Large diameter vs small diameter tool

This experiment was the same as Experiment 9, except that the crows were presented with a choice between two white tools 80 mm long with which they could probe the Perspex crevice. One stick had a larger diameter than the other, making it 3 times larger in volume and two times heavier in weight.

All experiments were done in the order described above with the following exceptions: Laura and Caesar were given Experiment 6 then Experiment 5, and Pepe and Mimic were given Experiment 10 then Experiment 9.

## Results

### Experiment 1: the Aesop's fable paradigm

None of the four tested crows dropped stones into the tube in their five trials with this problem, which indicated that they did not have *a priori* knowledge that putting stones into water would raise the water's level. However, they all learnt to drop stones into the tube from the platform. The number of times the crows needed to observe the effect of an accidental fall by a stone on the water level before they began actively dropping stones was low (mean ± s.e.m.: 12.25±6.9). The number of times a crow obtained the reward after an accidental drop was also low (mean ± s.e.m.: 4.25±2.29). Mimic began dropping stones after five accidental stone-drops, only one of which was rewarded; during training Bess showed a neophobic reaction to this apparatus and was replaced with Mimic. The reachable height for the five crows was established in 5.6±1.03 trials (mean ± s.e.m.).

### Experiment 2: matching number of stones to distance to water

All four crows solved the problem irrespective of the water level and never put in stones once they had removed the food. They also closely matched the required number of stones to the water level (stones dropped vs. distance to water; *R* ^2^ = 0.73, *F*_1,89_ = 236.4, *p*<0 .001).

### Experiment 3: Object discrimination; large vs. small stones

**Figure 3:**
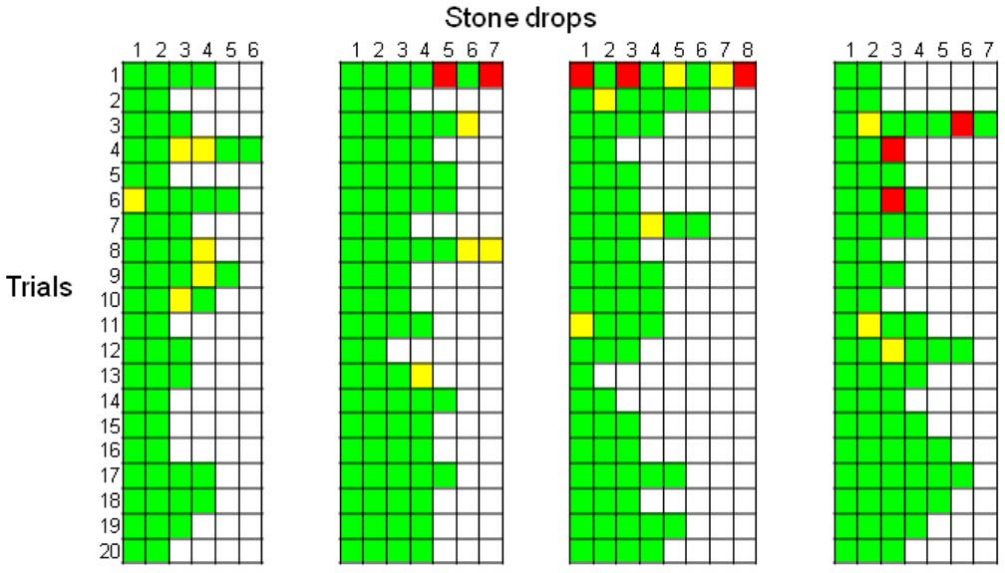
Trial-by-trial description of each individual stone drop in Experiment 3. Green squares indicate the drop of a large stone, red squares the drop of a small stone, and yellow squares indicates a small stone was picked up and discarded. Each column denotes the performances of one bird (from left to right: Laura, Caesar, Pepe, Mimic).

Across the 20 trials, the crows only dropped 8 small stones. Preference for the large stone was present after the first trial (binomial test, *p* = 0.019) and the first 5 trials (binomial test, *p*<0.001) (Figure 3). One crow, Laura, only ever dropped large stones. The crows also showed the distinctive behaviour of picking up and then discarding the small stone. Across the first 5 trials, the crows discarded a small stone 62% of the time that they picked one up. For two of the crows this occurred in the first trial before they had seen the effects of the small stone on the water level.

### Experiment 4: Substrate discrimination: sand vs. water

**Figure 4:**
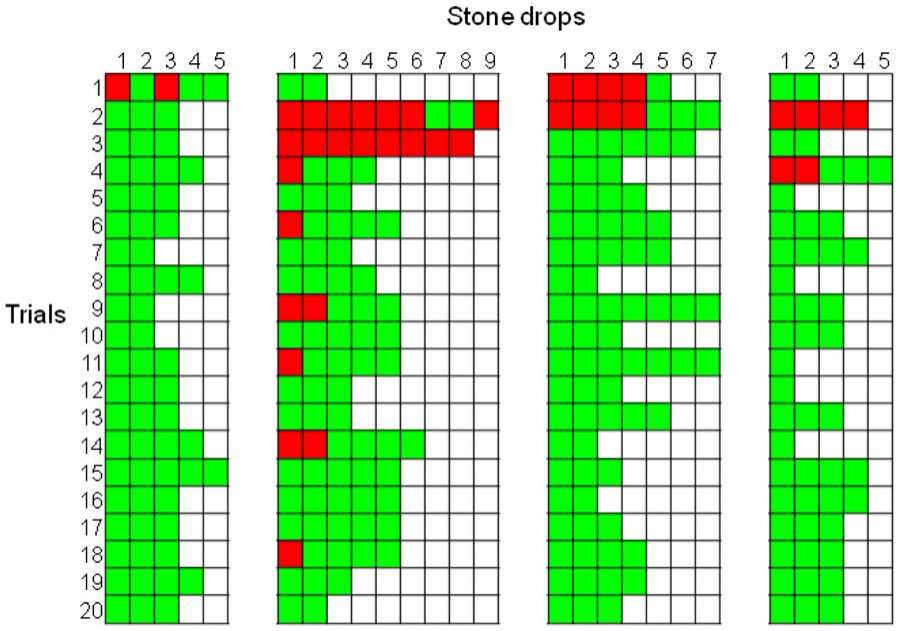
Trial-by-trial description of each individual stone drop in Experiment 4. Green squares indicate the drop of a stone into water, and red squares the drop of a stone into sand. Each column denotes the performances of one bird (from left to right: Laura, Caesar, Pepe, Mimic).

Across the first 5 trials, the crows dropped 61% of their stones into the water rather than the sand (binomial test, *p* = 0.048) (Figure 4). Laura performed above chance across the first 5 trials, dropping the stones into water rather than sand in 16 of her 18 drops (binomial test, *p* = 0.002) (see Laura's fourth trial in Movie S1).

### Experiment 5: Substrate discrimination: air vs. water

**Figure 5:**
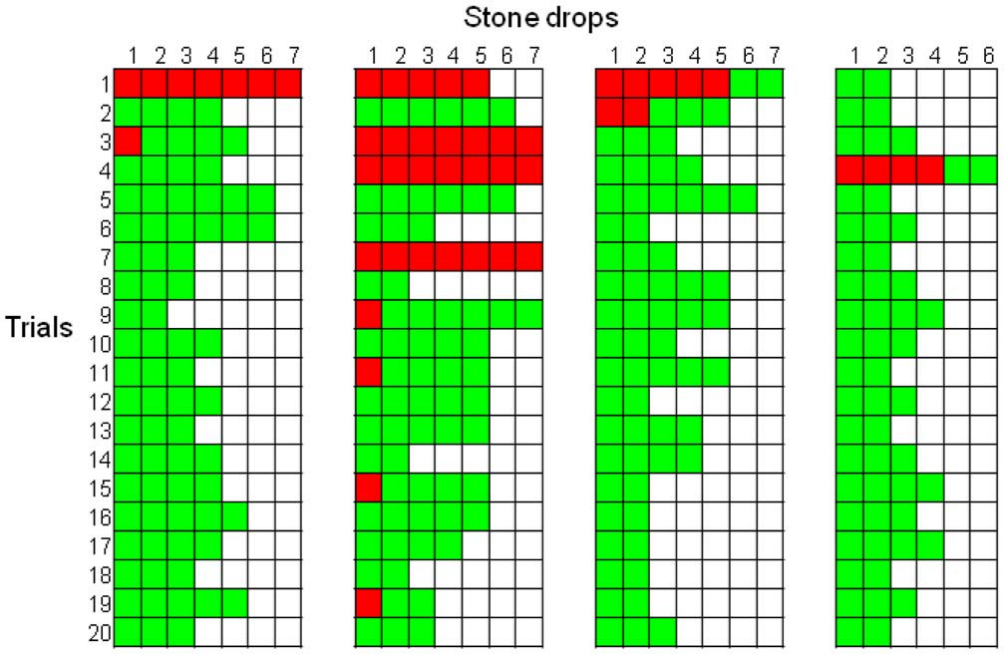
Trial-by-trial description of each individual stone drop in Experiment 5. Green squares indicate the drop of a stone dropped into water and red squares indicate the drop of a stone into air. Each column denotes the performances of one bird (from left to right: Laura, Caesar, Pepe, Mimic).

Across the first 5 trials, the crows dropped stones into water rather than air 60% of the time (binomial test, *p* = 0.042). However, in the first trial only 20% of the stone drops were into water (binomial test, *p* = 0.009) (Figure 5).

### Experiment 6: Object discrimination: heavy vs. light objects

**Figure 6:**
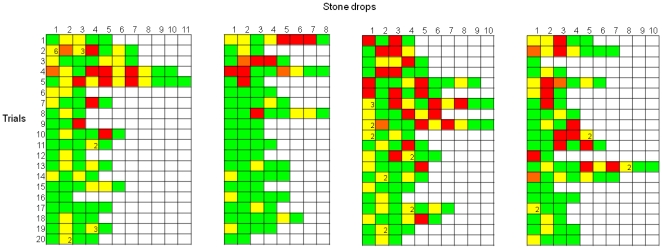
Trial-by-trial description of each individual object drop in Experiment 6. Green squares indicate the drop of a heavy block, red squares the drop of a light block, yellow squares the discard of a light block and orange squares the discard of a heavy block. Numbers within squares indicate the number of times that a light block was repeatedly picked up and discarded. Each column denotes the performances of one bird. (a) The performances of Laura (left) and Caesar (right). (b) The performances of Pepe (left) and Mimic (right).

Across the first 5 trials the crows chose to drop the rubber blocks 65% of the time (binomial test, *p* = 0.009) (Figure 6). On the first trial, the crows dropped nine rubber blocks and six polystyrene blocks, which was not significantly different from chance (binomial test, *p* = 0.60). However, the same discard behaviour that the crows had previously shown in the large/small stone experiment emerged again. On the first trial, the crows dropped the rubber blocks into the water every time that they picked them up, but dropped the polystyrene blocks into the water only 46% of the time that they picked them up (χ^2^_1_ = 4.83, *p* = 0.003). Across the first 5 trials this discrimination level remained similar – crows dropped the rubber blocks into the water 88% of the time that they picked them up and the polystyrene blocks 40% of the time (χ^2^_1_ = 25.76, *p*<0.0001). For three of the four crows, the first discard of a polystyrene block occurred before they had ever dropped blocks of this material into the water and observed how it interacted with water, namely that that it floated rather than sank (see Mimic's first trial in Movie S2).

### Experiment 7: Searching paradigm: large stone vs. small stone

An associative learning account predicts that the crows should treat the large stone as either a conditional reinforcer or a positive discriminative stimulus, and so search more in the tube next to this object. It also predicts that the crows should learn as quickly as in Experiment 3 that the large stone arbitrarily signalled success.

**Figure 7:**
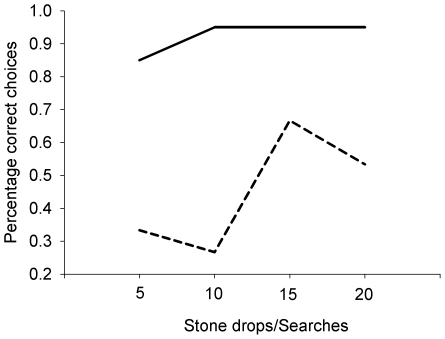
Learning curves for the large stone/small stone discrimination task during the stone dropping and searching paradigms. The solid line shows the learning curve for the stone dropping task (Experiment 3). The dashed line shows the learning curve for the searching task (Experiment 7).

The crows showed no preference to approach or search the tube with a large stone in front of it across the 20 trials. The crows chose the large stone in 46% of their 20 trials, even though they had chosen the large stone in 88% of their first 20 stone drops in the large/small stone Aesop's fable experiment (χ^2^_1_ = 6.13, *p* = 0.013) (Figure 7).

### Experiment 8: Searching paradigm: sand-filled tube vs. water-filled tube

The predictions for Experiment 8 were identical to those of Experiment 7: that crows should search more in the search-tube next to the water-filled tube because of past reinforcement history and learn as quickly as in Experiment 4 that the water-filled tube arbitrarily signalled success.

**Figure 8:**
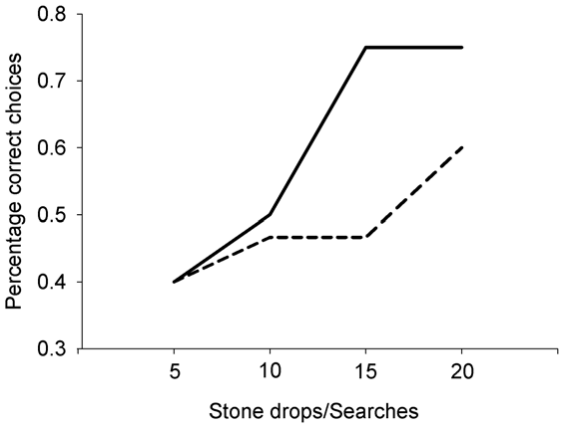
Learning curves for the water-filled tube/sand-filled tube discrimination task during the stone dropping and searching paradigms. The solid line shows the learning curve for the stone dropping task (Experiment 4). The dashed line shows the learning curve for the searching task (Experiment 8).

The crows showed no initial preference for the water-filled tube, nor did they learn across 20 trials to associate the water-filled tube with the reward. Comparison of correct responses between the first 20 stone drops of the Aesop's fable sand/water control and the 20 trials of this search task showed no overall difference in the proportion of correct choices (58% vs. 56%, respectively; χ^2^_1_ = 0.0003, *p* = 0.99). This non-significant result was due to the large inter-subject differences in the stone-dropping task across the first 20 drops, with Laura preferring water (18/20, binomial test, *p* = 0.0008) and Caesar preferring sand (15/20, binomial test, *p* = 0.044) (Figure 8). However, the proportion of correct responses that Laura made differed between the two experiments (χ^2^_1_ = 4.51, *p* = 0.034); she chose correctly in 90% of her first 20 stone drops compared to 55% of the time in her 20 search trials.

### Experiment 9: Tool discrimination: light vs. heavy tools

If crows had learnt a heuristic concerning the weight of objects, or merged their tool knowledge in a non-functional way, we predicted that they should continue to prefer heavy objects.

Across the first 5 trials of Experiment 9, the light and heavy tools were used equally often to probe the crevice (each tool was used for 46% of the 26 total probes; χ^2^_1_ = 0.77, *p* = 0.78), and the string tool was used for only 7% of the 26 probes (χ^2^_1_ = 7.93, *p* = 0.005). Furthermore, the three crows discarded the string in 8 of the 10 times it was picked up across these 5 trials. In the first trial, a similar pattern was seen - the light rigid tool was used 71% of the time and the heavy rigid tool was used 29% of the time (χ^2^_1_ = 1.075, *p* = 0.30) (the string was never used). The heavy rigid tool was discarded twice by the same crow, and the flexible light tool was also discarded twice but by two different crows. Three of the four crows initially picked up and discarded the string without using it in the crevice or pushing it against another object. Pepe was the only crow that first probed with the string before discarding it. He was also the only crow that did not discard the polystyrene block the first time he picked it up in Experiment 5.

### Experiment 10: Tool discrimination: large diameter tool vs. small diameter tool

We made the same predictions for Experiment 10 as we did for Experiment 9 – that the crows would prefer objects of larger volume and heavier weight, as they had done in the large stone/small stone dropping experiment. However, on the first trial three of the four crows chose the small diameter tool. Across the first 5 trials, 59% (N = 22) of the total probes were with the small diameter tool (binomial test, *p* = 0.52).

## Discussion

New Caledonian crows did not spontaneously use stones as tools by dropping them into the water-filled tube to bring floating food within reach. This indicates that the crows did not have *a priori* knowledge that dropping stones into the tube would raise the water level. However, after observing how stones falling into the water affected the water level and position of the floating food, the crows solved the task. Crucially, the crows also learnt about the functional properties of the stones and the tube contents. Crows showed an immediate preference for large, rather than small stones, with two crows actually discarding small stones the first time they picked them up and before they had observed their effect on the water level. The crows also had a preference after their first block of five trials to drop stones into water rather than sand, and to drop stones into water rather than an empty tube. When faced with heavy and light objects of the same size and colour, the crows showed a preference after five trials to drop heavy rather than light objects, with three crows discarding the light object when they first picked it up, before observing its effect on the water level. These discriminations show that the crows attended to the functional properties of both the object to be dropped and the substrate to be dropped into after very limited experience of dropping stones into water. These results are comparable to those with other corvids [13], [15].

The search paradigms examined learning when there was an arbitrary link between the property of an object or substrate and the reward. In these experiments, the crows had to search for food in a tube positioned next to a functionally relevant object or substrate. The crows did not learn across 20 trials to associate the previously rewarded object (large stone) or substrate (water-filled tube) with reward. This was despite these objects covarying with the outcome at the same level of consistency as in the stone dropping experiments and the crows having previously learned to prefer these same objects during the earlier experiments. An associative learning account predicts that learning should be guided by high levels of covariation between object and outcome. It also predicts that particular objects and tubes should become secondary or conditional reinforcers, due to their association with food. The lack of immediate preferences for the objects and the lack of learning of such a preference are not consistent with an associative learning account. In contrast, a causal learning account, where correlations between stimuli must also be causally relevant, can explain this difference. In this account, the association between large stones and food is functional in the stone-dropping task – large stones are positive stimuli because they raise the water level substantially more than small stones. In the search task the link between object and outcome is arbitrary – there is no reason why a large stone should signal the presence of hidden food. It therefore appears that arranging a causally appropriate relationship between object properties and food is either necessary for, or at least greatly facilitates, rapid learning.

In Experiments 9 and 10, the crows did not transfer the preferences for heavy or voluminous objects that they had formed during stone dropping to the stick tool tasks. Although New Caledonian crows can transfer information between perceptually distinct but structurally similar tasks (e.g. from the trap-tube to trap-table [17], [18]), they did not do this when the tasks were both perceptually and structurally different. That is, the mechanics of dropping stones into water to raise the water level were not the same as the mechanics of pulling food from a crevice. This indicates that even though the crows used stones as tools and learned about their functional properties, this knowledge did not affect, or merge with, their understanding of the functional properties of stick tools.

Our findings show that New Caledonian crows do not have a narrow-banded ability to process causal information that is restricted to sticks and stick-like objects and the interactions surrounding these items. Instead, this species quickly processes causal information about novel interactions between new tool types and the environment, without the acquisition of such information affecting their understanding of established tool behaviours. The differences between the causal and arbitrary tasks that we presented to the crows strongly suggest that cognitive mechanisms other than simple associative learning are involved in this processing of causal information. Investigating what these mechanisms are, and why the New Caledonian crow's tool use in the wild is highly context-specific, will be a focus of future work.

## Supporting Information

Movie S1 **Discrimination between sand and water (Experiment 4).** Fourth trial of Laura when faced with a tube filled with water and one filled with sand.
(MP4)

Movie S2 **Discrimination between heavy and light objects (Experiment 6).** First trial of Mimic when faced with light and heavy objects of the same size and colour.
(MP4)
